# Fluorescence Lifetime Imaging of NAD(P)H T Cells Autofluorescence in the Lymphatic Nodes to Assess the Effectiveness of Anti-CTLA-4 Immunotherapy

**DOI:** 10.17691/stm2023.15.3.01

**Published:** 2023-05-28

**Authors:** A.V. Izosimova, A.M. Mozherov, M.V. Shirmanova, V.I. Shcheslavskiy, D.A. Sachkova, E.V. Zagaynova, G.V. Sharonov, D.V. Yuzhakova

**Affiliations:** Laboratory Assistant, Laboratory of Genomics of Adaptive Antitumor Immunity, Research Institute of Experimental Oncology and Biomedical Technologies; Privolzhsky Research Medical University, 10/1 Minin and Pozharsky Square, Nizhny Novgorod, 603005, Russia; PhD Student, Department of Biophysics; National Research Lobachevsky State University of Nizhni Novgorod, 23 Prospekt Gagarina, Nizhny Novgorod, 603950, Russia;; Junior Researcher, Laboratory of Optical Spectroscopy and Microscopy, Research Institute of Experimental Oncology and Biomedical Technologies; Privolzhsky Research Medical University, 10/1 Minin and Pozharsky Square, Nizhny Novgorod, 603005, Russia;; Deputy Director for Science, Research Institute of Experimental Oncology and Biomedical Technologies; Privolzhsky Research Medical University, 10/1 Minin and Pozharsky Square, Nizhny Novgorod, 603005, Russia;; Head of the Laboratory of Optical Spectroscopy and Microscopy, Research Institute of Experimental Oncology and Biomedical Technologies; Privolzhsky Research Medical University, 10/1 Minin and Pozharsky Square, Nizhny Novgorod, 603005, Russia;; Master Student, Department of Biophysics; National Research Lobachevsky State University of Nizhni Novgorod, 23 Prospekt Gagarina, Nizhny Novgorod, 603950, Russia; Laboratory Assistant, Laboratory of Fluorescent Bioimaging, Research Institute of Experimental Oncology and Biomedical Technologies; Privolzhsky Research Medical University, 10/1 Minin and Pozharsky Square, Nizhny Novgorod, 603005, Russia;; Professor, Corresponding Member of the Russian Academy of Science, Leading Researcher, Laboratory of Optical Coherence Tomography, Research Institute of Experimental Oncology and Biomedical Technologies; Privolzhsky Research Medical University, 10/1 Minin and Pozharsky Square, Nizhny Novgorod, 603005, Russia;; Senior Researcher, Institute of Translational Medicine; Pirogov Russian National Research Medical University, 1 Ostrovitianova St., Moscow, 117997, Russia Senior Researcher, Laboratory of Genomics of Adaptive Antitumor Immunity, Research Institute of Experimental Oncology and Biomedical Technologies; Privolzhsky Research Medical University, 10/1 Minin and Pozharsky Square, Nizhny Novgorod, 603005, Russia;; Researcher, Laboratory of Genomics of Adaptive Antitumor Immunity, Research Institute of Experimental Oncology and Biomedical Technologies; Privolzhsky Research Medical University, 10/1 Minin and Pozharsky Square, Nizhny Novgorod, 603005, Russia;

**Keywords:** fluorescence lifetime imaging, FLIM, glioblastoma, metabolic status of tumor cells, NAD(P)H

## Abstract

The main problem in the field of tumor immunotherapy is the lack of reliable biomarkers that allow pre-determining the susceptibility of individual patients to treatment, as well as insufficient knowledge about the resistance mechanisms.

Biomarkers based on the autofluorescence of metabolic coenzymes in immune cells can become a powerful new predictor of early tumor response to treatment, whereas the optical FLIM method can be a tool to predict the effectiveness of immunotherapy, which allows preserving the spatial structure of the sample and obtaining results on the metabolic status of immune cells in real time.

**The aim of the study** is to conduct a metabolic autofluorescence imaging study of the NAD(P)H metabolic coenzyme in immune cells of freshly isolated lymph nodes as a potential marker for assessing the effectiveness of an early response to immunotherapy.

**Materials and Methods:**

The study was carried out on C57Bl/6 FoxP3-EGFP mice with B16F0 melanoma implanted near the inguinal lymph node. The mice were injected with antibodies to CTLA-4 (Bio X Cell, USA) (250 μg per mouse, intraperitoneally on days 7, 8, 11, and 12 of the tumor growth). FLIM images in the nicotinamide adenine dinucleotide (phosphate) coenzyme (NAD(P)H) channel (excitation — 375 nm, reception — 435–485 nm) were received using an LSM 880 fluorescent confocal laser scanning microscope (Carl Zeiss, Germany) equipped with a FLIM Simple-Tau module 152 TCSPC (Becker & Hickl GmbH, Germany). Flow cytometry was conducted using a BD FACSAria III cell sorter (BD Biosciences, USA).

**Results:**

Immunotherapy with checkpoint inhibitors resulted in marked metabolic rearrangements in T cells of freshly isolated lymph nodes in responder mice, with inhibition of the tumor growth. Fluorescence lifetime imaging data on NAD(P)H indicated an increase in the free fraction of NADH α_1_, a form associated with glycolysis to meet high demands of the activated T cells and pro-inflammatory cytokine synthesis. In contrast, non-responder mice with advanced tumors showed low values of the ratio of free fraction to bound α_1_/α_2_, which may be related to mechanisms of resistance to therapy.

The response to immunotherapy was verified by data on the expression of activation and proliferation markers by means of flow cytometry. The authors observed an increase in the production of the pro-inflammatory cytokine IFN-γ in effector T cells in responder mice compared to untreated controls and non-responders. In addition, an increase in the expression of the surface activation markers CD25 and CD69 was registered compared to untreated controls.

**Conclusion:**

Use of the FLIM method allowed to demonstrate that autofluorescence of the NAD(P)H coenzyme is sensitive to the response to checkpoint immunotherapy and can be used as a reliable marker of the effectiveness of response to treatment.

## Introduction

Cancer immunotherapy based on immune checkpoint inhibitors is one of the fastest growing focus areas of modern oncoimmunology and is highly effective in some patients with melanoma, non-small cell lung cancer, bladder cancer, breast cancer, and other forms of malignant neoformations. Currently, the following medications for two checkpoints are approved: CTLA-4 and PD-1 [1]. At that, despite the unprecedented clinic success of this therapy, a significant part of patients receiving medications of this group do not respond to treatment, which is due to individual characteristics of the patient’ immunity and peculiarities of the tumor interaction with the immune system [2, 3].

Globally, scientists search for prediction markers of immunotherapy with checkpoint inhibitors. Over the past decade, many biomarkers have been proposed to predict the effect of immunotherapy.

The predictors that are routinely used in selection of patients for treatment are a combination of the data from the study of the subpopulation composition of tumor-infiltrating lymphocytes and peripheral blood lymphocytes in combination with the level of expression of the CTLA-4 and PD-1/PD-L1 checkpoints. More complex and expensive methods include high-throughput sequencing based on transcriptome analysis of patient’s isolated lymphocytes with subsequent determination of their clonal composition, assessment of intratumoral mutation load, and determination of the allelic composition of the main histocompatibility complex [4-6].

However, these methods can not always accurately predict the therapy effectiveness immediately before treatment, they are extremely time-consuming, require expensive equipment and reagents, and provide delayed results, and thus currently, for the most common cancer types, the proportion of patients with a significant response to immunotherapy does not exceed 30%. In this regard, searching for reliable biomarkers remains relevant.

One of the most probable causes of resistance to immunotherapy with checkpoint inhibitors is the influence of the tumor microenvironment on the metabolic profile of T lymphocytes. For instance, the tumor environment can hinder implementation of the metabolic program of the effector T cells, competing with them for glucose, and, in contrast, attract regulatory and depleted T lymphocytes with excess lactate and fatty acids [7-9]. It is well-known that changes in metabolic processes precede the development of all subsequent events in immune cells (changes in expression profile, proliferation, etc.), and thus, the metabolic status of immune cells can potentially serve as a reliable prediction biomarker which is sensitive to early immunological rearrangements [1].

An innovative approach in cell metabolism studying is fluorescence lifetime imaging (FLIM), which is a highly sensitive and safe method to detect autofluorescence of metabolic cofactors in living cells. FLIM allows to quickly get the results of metabolism assessment and work in real time, does not require additional staining and intervention in physiological processes [10, 11].

Two coenzymes are targets for metabolic FLIM: reduced nicotinamide adenine dinucleotide (phosphate) (NAD(P)H) and oxidized flavin adenine dinucleotide (FAD); they participate in many biochemical reactions as electron carriers. The analysis of the metabolic status by the coenzymes fluorescence lifetime is based on the fact that their fluorescence lifetime significantly depends on the microenvironment, interaction with proteins, and their conformation [12, 13].

Autofluorescence imaging is widely used to study metabolism of cancer and other diseases [14, 15]. However, its application as a means to analyze the functional state of immune cells began to develop only a short time ago. Currently, there are only a few works on optical metabolic imaging of immune cells published. In 2020–2022, the results of metabolic FLIM of macrophages [16-18] and T lymphocytes studies were published [19, 20]. The ability of metabolic imaging to distinguish between active T cells and T cells in rest *in vitro*, as well as various subpopulations of innate and adaptive immunity cells was demonstrated [19, 20]. However, subpopulations of T cells in these studies were isolated from healthy donors and cultured *in vitro*, whereas various states of activation were artificially modeled by adding non-antigenic stimuli. Until fairly recently, there were no publications on metabolic imaging of T cells of donors with oncology.

In 2022, we originally developed a protocol for metabolic imaging of T cells in fresh lymphoid tissue, that is under almost natural conditions [21]. Using a mouse melanoma model, it was demonstrated for the first time that NAD(P)H autofluorescence parameters in lymphocytes were sensitive to tumor development and could act as a reliable biomarker for assessing the immune response to a tumor.

**The aim of the study** is to validate the method of metabolic fluorescence lifetime imaging to solve the problem of predicting the effectiveness of immunotherapy on freshly isolated lymphocytes.

It is known that melanoma is one of the main targets for immunotherapy with checkpoint inhibitors due to its high mutation load, which contributes to its high immunogenicity [1, 22], thus the study was carried out on C57Bl/6 FoxP3-EGFP mice with B16F0 melanoma. Experiments were conducted on freshly isolated lymph nodes on days 13–14 after tumor implantation. As metabolic changes are the earliest events in immune cells in response to treatment, lymph nodes were sampled 1–2 days after the last injection of the therapeutic antibody. This early period was chosen based on our previous studies [23-25]. Activation and proliferation of effector T cells were confirmed by flow cytometry.

## Materials and Methods

### Cell cultivation

 B16F0 mouse melanoma tumor cells were cultured with the standard method on RPMI nutrient medium (Roswell Park Memorial Institute medium; Gibco, USA) added with 0.06% L-glutamine, 10% fetal bovine serum (Gibco, USA), 50 U/ml of penicillin, and 50 μg/ml of streptomycin sulfate (PanEco, Russia) in culture flasks (25 cm^3^) (Corning, USA) in an incubator at 5% CO_2_ at 37°C and 85% humidity. Cells were removed using a 25% trypsin–EDTA solution (PanEco, Russia) during 5 min.

### Tumor model

 Objects of the study were mice of a special transgenic FoxP3-EGFP line based on the C57BL/6 line, with the chimeric factor FoxP3 associated with the green fluorescent protein EGFP expressed in regulatory T lymphocytes for specific labeling.

Experiments were conducted on 43 C57BL/6-FoxP3-EGFP transgenic mice (kindly provided by Alexander Rudensky, Sloan Kettering Institute, New York, USA). To identify regulatory FoxP3+CD4+T cells in these mice, a chimeric EGFP construct subcloned into the first exon of the *FoxP3* gene [26] was knocked out.

To obtain a tumor model, the mice (females, 2–4 months old) were injected with a suspension of tumor cells at a dose of 100,000 cells in 150 μl of phosphate-buffered saline (PBS). The injection was conducted subcutaneously closer to the large and easily found inguinal lymph node, the animal was lying on its side.

During all surgical procedures, the mice were anesthetized intramuscularly with a mixture of Zoletil — 40 mg/kg, 50 μl (Virbac France S.A., France) and 2% Xylazine — 10 mg/kg, 10 μl (Interchemi Werker De Adelaar Esti AS, Estonia). All animal studies are approved by the local ethics committee of the Privolzhsky Research Medical University (Nizhny Novgorod, Russia) (protocol No.12 of August 5, 2022).

### Anti-CTLA-4 immunotherapy

 The mice were injected with antibodies to CTLA-4 (Bio X Cell, USA) (250 μg per mouse, intraperitoneally, on days 7, 8, 11, and 12 of the tumor growth). This treatment regimen was chosen based on our previous studies [21, 23–25].

Untreated mice with a tumor were taken as a control group. Tumor node growth was monitored to determine responder and non-responder mice. The tumor size was determined by using two measurements of a vernier caliper for subsequent calculation of the volume according to the following formula: *V= a***·***b***·***b*/2, where *a* is the length and *b* is the width of the tumor node.

Measurements were taken every 1–2 days, starting from day 5 after tumor cell implantation and ending on day 13–14, when the inguinal lymph node was sampled. With this purpose, the mice were euthanized with 90% Isoflurane (Laboratories Karizoo, Spain). Lymph nodes were excised immediately after mice death using microsurgical scissors and Leica M50 stereomicroscope (Leica Microsystems, Germany), and then divided into two parts: for FLIM and for flow cytometry, respectively.

### Fluorescence lifetime imaging

 Using the earlier developed protocol [21], metabolic visualization of T cells in fresh lymphoid tissue was conducted. A fragment of a lymph node (proportion of ~1/3 and thickness of ~1 mm) was cut off with microsurgical scissors and placed with the cut down on a FluoroDish glass bottom dish (WPI, China) so that the lymph node capsule did not block the fluorescent signal from the immune cells. The fragment of the lymph node was covered with a cloth moistened with 0.9% saline to avoid drying and displacement of the sample during microscopy. The procedure duration from the isolation of the lymph node to the end of the experiment did not exceed 20 min.

Visualization was conducted using an LSM 880 laser scanning microscope (Carl Zeiss, Germany). The femtosecond Τi:Sa laser (Spectra Physics, USA) with a pulse repetition rate of 80 MHz and a duration of 120 fs with a tunable wavelength in the range of 690–1040 nm was used as the excitation source. Fluorescence lifetime detection was carried out by using a Simple-Tau 152 ΤCSPC FLIM module (Becker & Hickl GmbH, Germany) based on the time-correlated single photon counting. Images were obtained by using a C Plan-Apochrom at 40x/1.3 NA Oil DIC M27 oil immersion objective (Carl Zeiss, Germany). Excitation of the fluorescence of the NAD(P)H metabolic coenzyme was conducted in a two-photon mode at a wavelength of 750 nm and received in the range of 450–490 nm. The power of the exciting radiation was 7 mW. The number of photons per pixel was at least 5000 with binning 3, the signal accumulation time was 60 s.

Single-photon EGFP fluorescence was excited at a wavelength of 488 nm, and emission was recorded in the range of 500–570 nm.

Fluorescence lifetime imaging data were processed by means of SPCImage software (Becker & Hickl GmbH, Germany). To determine the decay curves parameters in each pixel, the authors used the least squares approximation.

In case of NAD(P)H, the short lifetime component corresponded to its free glycolysis-associated form, whereas the long component corresponded to the protein-bound form of NAD(P)H associated with the mitochondrial electron transport chain and involved in oxidative phosphorylation.

The fluorescent decay curves were approximated by means of a bi-exponential model, which allows to determine the short and long components (τ_1_ and τ_2_, respectively) and the relative amplitudes of these components (α_1_ and α_2_, respectively). These values were used to determine the amplitude-weighted average fluorescence lifetime by using the following formula: τ*_m_*=α_1_τ_1_+α_2_τ_2_. The approximation accuracy was assessed by using the parameter χ^2^. For all data, χ^2^ was in the range of 0.8–1.2.

The image was imported into the running software, areas with a valid value of χ^2^ were selected, and the area of the cell cytoplasm was specified, excluding the cell nucleus. For each lymph node, 2–3 visual fields were identified with a total of 40–60 cells.

### Flow cytometry

 To assess the impact of therapy on the expression of the major activation and proliferation markers, a fragment of a lymph node was mechanically disaggregated in 100 μl of PBS. Live cells were labeled with antibodies to CD3, CD4, CD8, CD25, and CD69 receptors (BD Biosciences, USA). Intracellular staining was conducted with the cells fixed, permeabilized, and stained with CD3, CD4, CD8 antibodies (BD Biosciences, USA) and IFN-γ (Miltenyi Biotec, Germany) in line with the recommendations of the Inside Stain Kit manufacturer (Miltenyi Biotec, Germany). Labeled cells were analyzed on a BD FACSAria III cell sorter (BD Biosciences, USA). 300,000 cells were sampled from each lymph node. Data were analyzed by means of the FlowJo software (Tree Star, USA).

### Statistical analysis

 The comparative data analysis and graphic visualization were conducted by means of the GraphPad Prism 8.0.1 software (GraphPad Software, USA). To analyze the rate of tumor growth, the average tumor volume was calculated for all mice in each experimental group on a specific day. Data were taken as a mean ± standard error of the mean (M±SEM). The statistically significant differences between the control and anti-CTLA-4 treated study groups were calculated using the nonparametric Mann–Whitney test (the differences were considered statistically significant at p≤0.05).

Continuous variables were tested for normality of distribution by using the Shapiro–Wilk test (the distribution was considered normal at p≤0.05).

The FLIM data were analyzed by calculating the average value of each parameter for 40–60 cells (2–3 visual fields) in the lymph node of each mouse. Then, the average value for all mice was calculated in each experimental group. The statistically significant differences between the control, responders in anti-CTLA-4-treated, and non-responders in anti-CTLA-4-treated study groups were determined by using the nonparametric Mann–Whitney test (differences were considered statistically significant at p≤0.05).

The analysis of flow cytometry data was conducted by using 300,000 cells which were collected from each lymph node on a cell sorter. The assessment related to the percentage of cells expressing a certain marker from the total number of cells in a particular subpopulation of T lymphocytes. An average value was calculated for all mice in each experimental group. The statistically significant differences between the control, responders in anti-CTLA-4-treated, and non-responders in anti-CTLA-4-treated study groups were determined by using the nonparametric Mann–Whitney test (differences were considered statistically significant at p≤0.05).

## Results

### Monitoring of the tumor growth in mice

 At the first stage of the study, the monitoring of the tumor growth in mice from the control and anti-CTLA-4-therapy groups was conducted up to the day of the lymph node sampling for FLIM and flow cytometry.

**Figure 1.**
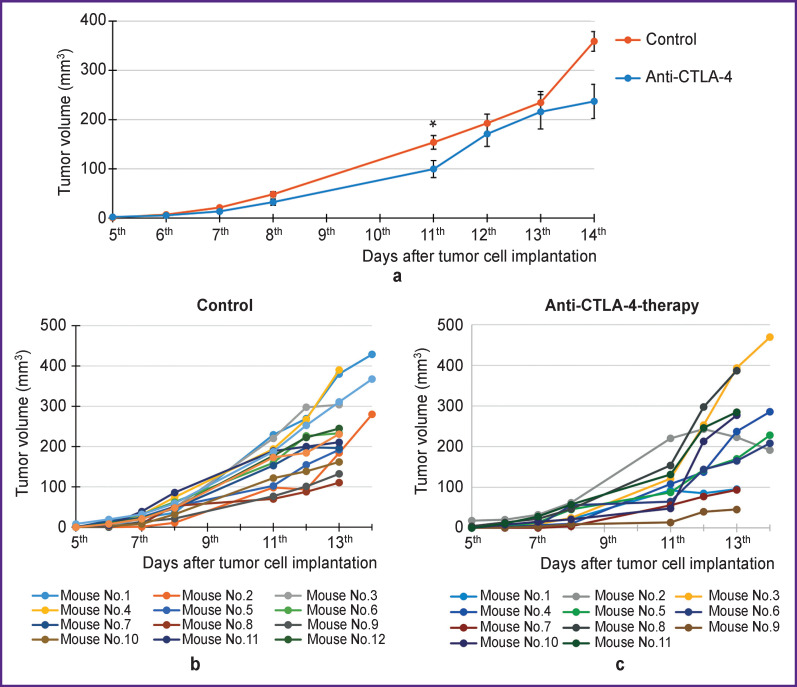
Monitoring of tumor growth in mice during anti-CTLA-4 immunotherapy: (a) the average tumor growth rate curves of the control and anti-CTLA-4-therapy groups; data are shown as the mean ± standard error of the mean; * is the statistically significant difference between groups on day 11 of the tumor growth, p=0.0211, nonparametric Mann–Whitney test; (b), (c) individual tumor growth curves for each mouse in the control and anti-CTLA-4-therapy groups, respectively

Analysis of the average tumor growth rate curves in the control and anti-CTLA-4-therapy groups (Figure 1 (a)) showed that, in general, the anti-CTLA-4-therapy group demonstrated a trend towards a decrease in tumor growth rate compared to the control group. However, statistically significant differences were seen only on day 11 of the tumor growth.

It is known that melanoma is characterized by high immunogenicity and due to it there is a divergence in values of the individual tumors growth rate (Figure 1 (b)). During treatment, this divergence becomes more pronounced due to heterogeneous response to immunotherapy (Figure 1 (c)). Thus, on the day of the lymph node sampling, the volume of the tumor node in the anti-CTLA-4-therapy group varied from 45 to 470 mm^3^, whereas in the control group the variations were less pronounced — from 110 to 380 mm^3^. In the treatment group, it was possible to distinguish both mice with a pronounced inhibition of the tumor growth, and mice with a growth rate comparable to the control tumors or higher.

Thus, the selected scheme of immunotherapy led to a result similar (according to the clinical statistics) to the situation when the maximum effect is achieved in maximum 30% of patients, and allowed to identify responder and non-responder mice.

### Fluorescence lifetime imaging of NAD(P)N

 The next stage of the study was devoted to assessment of the autofluorescence of the NAD(P)H metabolic coenzyme in the immune cells of the mice lymph nodes. The values of the lifetimes of the short and long components τ_1_ and τ_2_, as well as their relative contributions α_1_ and α_2_ and the NAD(P)H average lifetime τ*_m_* in T cells which were averaged for each lymph node.

The absolute values of the NAD(P)H lifetimes recorded in the immune cells for both groups corresponded to the standard values reported in the literature [10, 27] (see the Table). In the control and anti-CTLA-4-therapy groups, the lifetime of the free form (τ_1_) was 0.48±0.01 and 0.49±0.01 ns, whereas of the protein-bound form (τ_2_) was 2.58±0.08 and 2.53±0.08 ns, respectively. The difference in the values of the absolute lifetimes between the groups was not statistically significant.

**Table:** Fluorescence lifetime of NAD(P)H coenzyme in immune cells of lymph nodes of mice with tumor (M±SEM)

Group	τ**_1_ (ns)**	τ**_2_ (ns)**
Control	0.48±0.01	2.58±0.08
Anti-CTLA-4-immunotherapy	0.49±0.01	2.53±0.08

**Figure 2.**
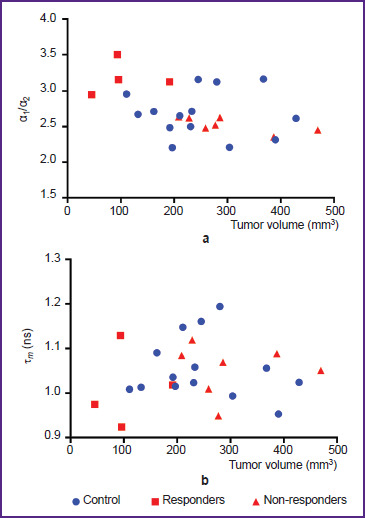
Scatter charts of the distribution of α_1_/α_2_ (a) and τ*^m^* (b) NAD(P)H values depending on the tumor volume on the day of lymph node sampling Each point corresponds to the average value for the lymph node cells of each mouse

To assess the ratio of the α_1_/α_2_ contributions and the τ*_m_* mean lifetime, we created distribution diagrams of these values depending on the tumor volume on the day of the lymph node sampling (Figure 2 (a)).

It was established that the α_1_/α_2_ parameter demonstrated a correlation with the tumor volume. It was found that mice with the smallest tumor volume after anti-CTLA-4 therapy (45–95 mm^3^, mice No.1, 7, and 9, see Figure 1 (c)) showed the highest values of α_1_/α_2_ compared with other mice both with treatment and without it. Moreover, a high value of α_1_/α_2_ was also shown by mouse No.2 from the anti-CTLA-4-therapy group (the tumor volume of 191 mm3), which, despite the initially large tumor, demonstrated the tumor growth inhibition starting from day 11 and reduction in the tumor node size.

On the contrary, those mice which, despite therapy, had a progressing large tumor with a volume of 208– 470 mm3, showed low α_1_/α_2_ values comparable to those of the control mice without therapy.

Based on the data received, we could distinguish two subgroups of mice in the anti-CTLA-4-therapy group: responders (mice No.1, 2, 7, and 9) (red squares in Figure 2) and non-responders (mice No.3, 4, 5, 6, 8, 10, 11) (red triangulars in Figure 2).

There was no such a clear dependence for τ*_m_* (Figure 2 (b)). However, specific responder mice (No.1 and 9) showed the lowest τ*_m_* values. In the rest of the mice, the values of τ*_m_* after treatment did not differ from the control group.

**Figure 3.**
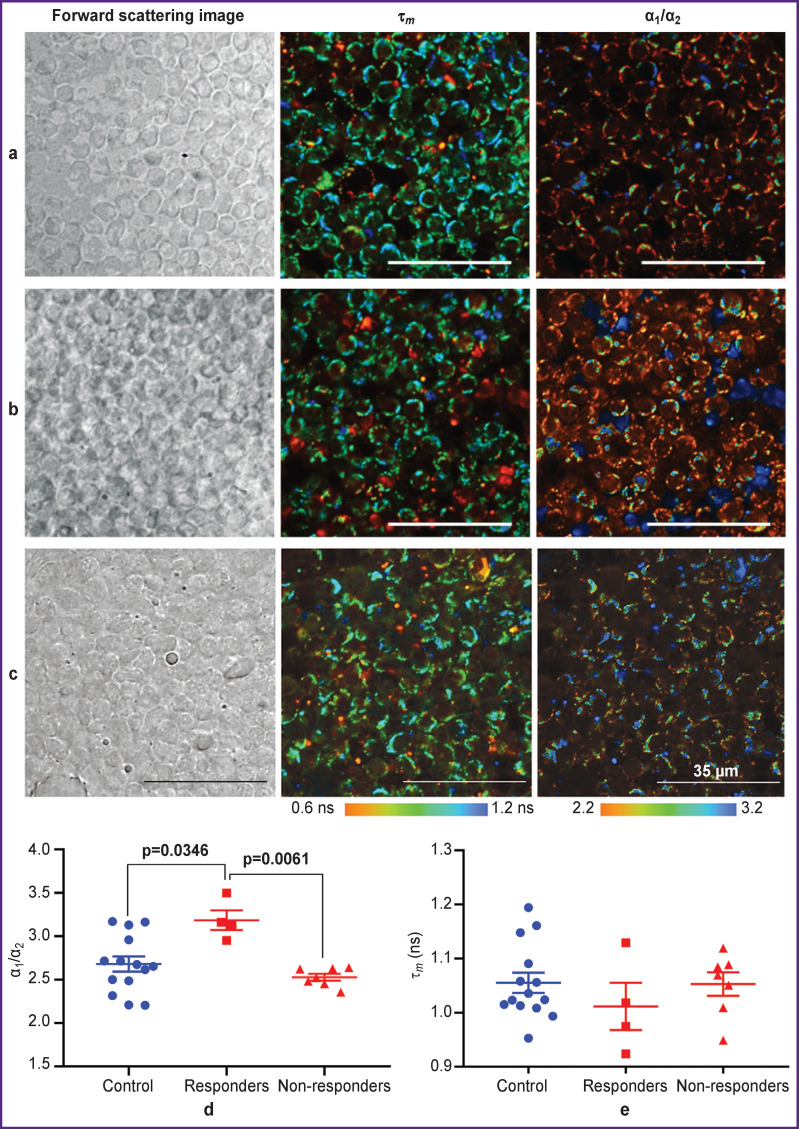
Fluorescence lifetime imaging of NAD(P)H in immune cells of the lymph nodes in mice with tumor Representative FLIM images of τ*^m^* and α_1_/α_2_ NAD(P)H in the lymph nodes of mice from the following groups: control (a), anti-CTLA-4-therapy/non-responders (b), and anti-CTLA-4-therapy/ responders (c); scatter charts of α_1_/α_2_ (d) and τ*^m^* (e) NAD(P)H values in these groups. Horizontal lines correspond to the group mean and the standard error of the mean

For statistical processing, scatter charts were prepared which illustrate the α_1_/α_2_ and τ*_m_* values for the control, anti-CTLA-4-therapy/responders, and anti-CTLA-4-therapy/non-responders groups of mice (Figure 3).

It was established that the average α_1_/α_2_ ratio in the anti-CTLA-4-therapy/responders group was 3.18±0.11, which was statistically significantly higher compared to the value of 2.68±0.08 in the control group (p=0.0346) and with the value of 2.52±0.04 in the anti-CTLA-4-therapy/non-responders group (p=0.0061) (Figure 3 (d)).

No significant differences were seen between the control and non-responder mice groups.

The τ*_m_* parameter showed a downward trend in the responders group due to an increase in the contribution of the free (short) component without statistical significance (Figure 3 (e)).

Thus, the α_1_/α_2_ parameter can be used as a marker of the effectiveness of the response to immunotherapy with checkpoint inhibitors. An increase in the contribution of the free form and, correspondingly, a decrease in the contribution of the protein-bound form of NAD(P)H may be associated with a shift in the cellular metabolism towards glycolysis.

### Expression of activation and proliferation markers by effector T cells

 To verify the response to immunotherapy, we assessed the activation of effector T cells in two main subpopulations: cytotoxic CD8+ T lymphocytes and helper CD4+ T lymphocytes (CD4+Th) isolated from the lymph nodes.

**Figure 4.**
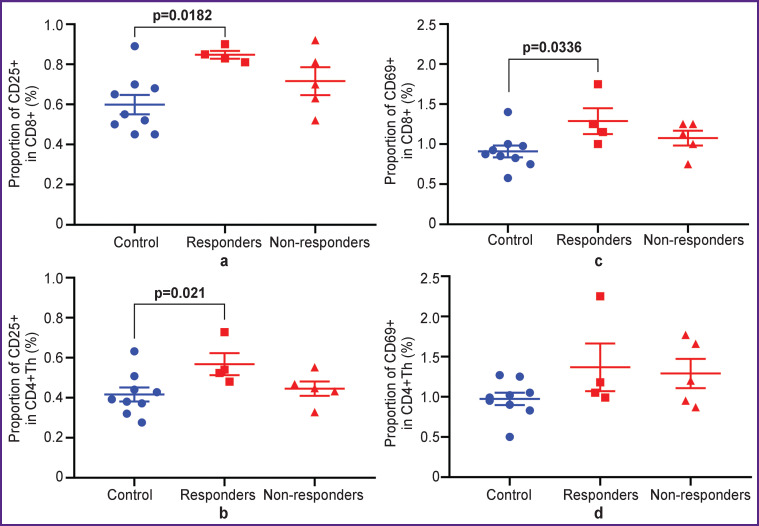
Analysis of expression of activation markers CD25 (a, b) and CD69 (c, d) in live CD8+ and CD4+Th T cells, respectively Scatter charts show measurements for individual animals (*dots*) and the group mean and standard error of the mean (*horizontal lines*)

At first, we analyzed the expression of the surface markers of early (CD69) and intermediate (CD25 (IL-2Rα)) activation in the live immune cells (Figure 4). There was a statistically significant increase in the proportion of the activated cells after therapy in responder mice compared to the control group. Flow cytometry data showed an increase in the proportion of CD25+ cells in the subpopulations of CD8+ (p=0.0182) and CD4+Th (p=0.021) T lymphocytes, as well as in the proportion of CD69+ cells among CD8+ T cells (p=0.0336). The anti-CTLA-4-therapy/non-responders group did not differ in the expression of the surface activation markers from the anti-CTLA-4-therapy/ responders and the control group.

**Figure 5.**
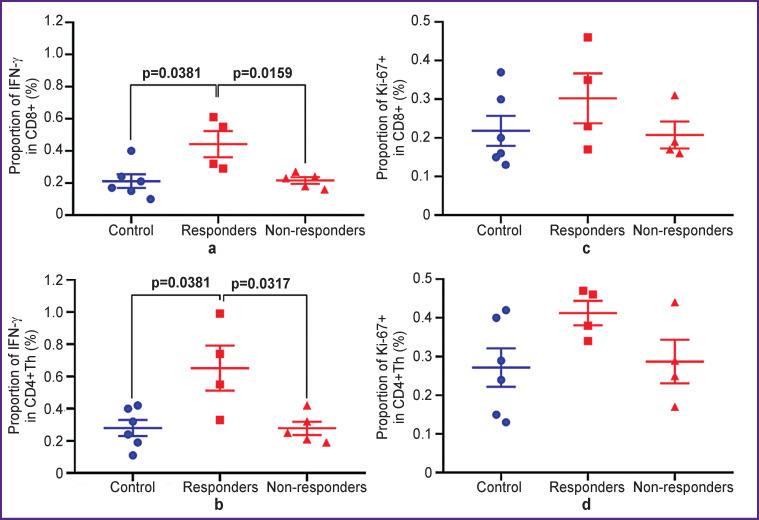
Analysis of IFN-γ production (a, b) and expression of the proliferation marker Ki-67 (c, d) in the fixed CD8+ and CD4+Th T cells, respectively Scatter charts show measurements for individual animals (*dots*) and the group mean and standard error of the mean (*horizontal lines*)

Assessment of intracellular expression of the pro-inflammatory cytokine IFN-γ as a marker of early activation and proliferation marker Ki-67 by using intracellular staining of fixed immune cells (Figure 5) showed a statistically significant increase in the level of IFN-γ production in responder mice compared with the control and non-responder mice. An increase in the proportion of IFN-γ+ cells was seen in both subpopulations of effector lymphocytes CD8+ (p=0.0381 and p=0.0159) and CD4+Th (p=0.0381 and p=0.0317) compared with the control and anti-CTLA-4-therapy/ non-responders groups, respectively.

The Ki-67 proliferation index did not demonstrate statistically significant differences between the groups. At that, there was a non-significant trend towards an increase in the proportion of Ki-67+ cells in responder mice, which was more pronounced in the CD4+Th subpopulation.

Thus, the effector T cells, as expected, were activated in response to immunotherapy, and the expression of the main activation markers was increased only in the responder mice group. As the selected time of the lymph node sampling corresponded to an early response to therapy, we noted a difference between responders and non-responders only in the production of IFN-γ+ as the earliest marker of activation. For this period, there was either no significant increase in the immune cell proliferation, which is typical for the later stages after treatment [23-25].

## Discussion

The key problem in immunotherapy with tumor checkpoint inhibitors is the lack of reliable biomarkers that allow predetermining the susceptibility of specific patients to treatment, as well as insufficient knowledge about the mechanisms of resistance [1]. Biomarkers on basis of the autofluorescence of metabolic coenzymes can become a new powerful predictor of the early tumor response to treatment, whereas the optical FLIM method can become a tool to predict the effectiveness of immunotherapy, which allows preserving the spatial structure of the sample and fast receiving and processing of the data on the metabolic status of immune cells.

In this study, we studied the autofluorescence of immune cells in fresh fragments of the lymph nodes during anti-CTLA-4 therapy of mice with B16F0 melanoma at an early stage of treatment by means of the FLIM method. Metabolic imaging of immune cells during immunotherapy was conducted for the first time.

The NAD(P)H coenzyme lifetime parameters were chosen as the target for metabolic imaging. In our earlier publication [21], we showed that these particular parameters demonstrate sensitivity to tumor development. We evaluated how tumor progression leads to a gradual increase in the glycolysis-associated NADH α_1_ free form and development of the NADPH α_3_ phosphorylated component associated with biosynthetic processes; these phenomena reflect an increase in the antitumor immune response via the increasing release of tumor antigens. Flow cytometry data confirmed that changes in the free NADH proportion correlated with the activation of CD4+Th and CD8+ T cells, whereas changes in the bound NADPH correlated with proliferation of the mentioned cells [21].

This study established that the NADH α_1_/α_2_ parameter was sensitive to response to immunotherapy with checkpoint inhibitors.

The increase in the NADH α_1_ free form contribution and a corresponding decrease in the contribution of the NADH α_2_ protein-bound form correlate with a positive response to anti-CTLA-4 therapy expressed in the tumor growth inhibition. An increase in α_1_/α_2_ in responders may be associated with a shift in the cellular metabolism towards glycolysis.

It is known that the CTLA-4 inhibitory receptor on T cells mainly hinders normal antigen presentation by antigen-presenting cells (APC) by competing with the CD28 receptors for binding to the B7 ligands (B7-1/CD80 and B7-2/CD86) to APC and depriving T cells of a co-stimulating signal [28, 29]. Moreover, this receptor represses the PGC-1α transcriptional coactivator, which results in the impaired metabolic shift towards glycolysis [30]. Blockade of CTLA-4 allows the metabolic program of the effector T cells to implement in the draining lymph nodes [31]. Normal stimulation of the T-cell antigen receptor results in activation of the phosphatidylinositol-3-kinase (PI3K)/ Akt/mTORC1 signaling pathway and Myc induction, which contributes to an increase in the aerobic glycolysis and glutaminolysis in order to support high energy and biosynthetic demands of the cell [30, 31], and this finding is consistent with the results of the study.

However, we registered such a positive response only in 36% of mice, which is consistent with the clinical statistics, when the maximum effect of checkpoint therapy is achieved in maximum 30% of patients [1, 32]. Non-responder animals showed low α_1_/α_2_ values comparable to those of the untreated control mice, which may be related to the disruption of the metabolic pathways responsible for switching to glycolysis.

The cause for this dysfunction can include the mechanisms of resistance described below. It is known that treatment with the blocking anti-CTLA-4 or anti-PD-1 antibodies can induce activation of other inhibitory receptors such as TIM-3, LAG-3, and VISTA, which in their turn also suppress glycolytic pathways in effector cells. Moreover, melanoma cells maintain the high glycolytic profile by activating the MAPK and (PI3K)/ Akt/mTORC1 signaling pathways (by means of V600E mutation activation in *BRAF* gene or *PTEN* inactivation) by competing with effector T cells for glucose. The tumor surroundings with excess lactate and fatty acids create a metabolic barrier for glycolytic effector cells and simultaneously promotes stability of regulatory T cells, which limits the effectiveness of anti-CTLA-4 checkpoint therapy [7, 33, 34].

The response to immunotherapy was verified by data on expression of activation and proliferation markers by means of flow cytometry. The authors recorded an increase in the expression of activation markers CD25, CD69, and IFN-γ in effector T cells in responder mice. At that, only the expression of IFN-γ being the earliest marker allowed to separate responders and non-responders. This phenomenon correlates with FLIM data on increase in the contribution of the free α_1_ component associated with glycolysis in responders. Aerobic glycolysis is known to promote the production of inflammatory cytokines such as IFN-γ. It maintains acetyl-CoA substrate pools that are required for epigenetic stimulation of the IFN-γ gene expression and enhances the IFN-γ mRNA translation into protein [8, 35].

In contrast to the previous study [21], no increase in τ_2_ and increase in the contribution of the phosphorylated form of NADPH α_3_ associated with biosynthetic processes and proliferation were noted in this study. Flow cytometry data on the expression of the proliferation marker Ki-67 correlate with the FLIM data, demonstrating the lack of a statistically significant difference between the studied groups. The most probable reason for that is the earlier development of the tumor and the early stage of therapy in this study.

Therefore, immunotherapy with checkpoint inhibitors leads to a significant reprogramming of T-cell metabolism. The data received by means of the FLIM study of NAD(P)H, namely the increase in α_1_/α_2_, reflect the development of an antitumor immune response as a reaction to checkpoint therapy in responder animals. Metabolic FLIM is more sensitive to individual changes at the early stages of treatment than the level of cell proliferation and expression of surface activation receptors.

## Conclusion

The study demonstrated that immunotherapy with checkpoint inhibitors results in pronounced metabolic rearrangements in T cells of freshly isolated lymph nodes in responder mice with inhibition of the tumor growth. Data on NAD(P)H fluorescence lifetime imaging showed an increase in the free fraction of the glycolysis-associated NADH α_1_ form to meet the high demands of activated T cells and synthesis of pro-inflammatory cytokines. On the contrary, non-responder mice with advanced tumors show low α_1_/α_2_ values, which may be related to treatment resistance mechanisms. FLIM data correlated with flow cytometry results for expression of activation markers and IFN-γ production.

The results received prove that the NAD(P)H coenzyme autofluorescence can be used as a reliable marker of the effectiveness of the response to checkpoint immunotherapy.
